# Chronic prehepatic portal hypertension in the rat: is it a type of Metabolic Inflammatory Syndrome?

**DOI:** 10.1186/1476-511X-7-4

**Published:** 2008-02-13

**Authors:** Fernando Sánchez-Patán, Raquel Anchuelo, Maria-Angeles Aller, Elena Vara, Cruz García, Maria-Paz Nava, Jaime Arias

**Affiliations:** 1 Surgery I Department, School of Medicine, Complutense University of Madrid, Spain; 2 Biochemistry and Molecular Biology III Department, School of Medicine, Complutense University of Madrid, Spain; 3 Department of Physiology (Animal Physiology II), School of Biology, Complutense University of Madrid, Spain

## Abstract

**Background:**

A progressive development of hepatic steatosis with an increase in the lipid hepatocyte content and the formation of megamitochondria have been demonstrated in rats with prehepatic portal hypertension. The aim of this study is to verify the existence of liver and serum lipid metabolism impairments in rats with long-term (2 years) portal hypertension.

**Methods:**

Male Wistar rats: Control (n = 10) and with prehepatic portal hypertension by triple partial portal vein ligation (n = 9) were used. Liver content of Triglycerides (TG), phospholipids (PL) and cholesterol and serum cholesterol, lipoproteins (HDL and LDL), TG, glucose and Lipid Binding Protein (LBP) were assayed with specific colorimetric commercial kits. Serum levels of insulin and somatostatin were assayed by RIA.

**Results:**

The liver content of TG (6.30 ± 1.95 *vs*. 4.17 ± 0.59 μg/ml; p < 0.01) and cholesterol (1.48 ± 0.15 *vs*. 1.10 ± 0.13 μg/ml; p < 0.001) increased in rats with portal hypertension. The serum levels of cholesterol (97.00+26.02 *vs*. 114.78 ± 37.72 mg/dl), TG (153.41 ± 80.39 *vs*. 324.39 ± 134.9 mg/dl; p < 0.01), HDL (20.45 ± 5.14 *vs*. 55.15 ± 17.47 mg/dl; p < 0.001) and somatostatin (1.32 ± 0.31 *vs*. 1.59 +0.37 mg/dl) decreased, whereas LDL (37.83 ± 15.39 *vs*. 16.77 ± 6.81 mg/dl; p < 0.001) and LBP (308.47 ± 194.53 *vs*. 60.27 ± 42.96 ng/ml; p < 0.001) increased.

**Conclusion:**

Portal hypertension in the rat presents changes in the lipid and carbohydrate metabolisms similar to those produced in chronic inflammatory conditions and sepsis in humans. These underlying alterations could be involved in the development of hepatic steatosis and, therefore, in those described in the metabolic syndrome in humans.

## Background

Portal Hypertension (PH) is a clinical syndrome defined by a pathological increase in blood pressure in the portal system [1,2]. It is one of the most frequent and serious complications of chronic liver disease [1,2]. In prehepatic PH there is no underlying liver disease [3] and thus its complications are related to the obstruction to the portal flow [1,2]. These alterations are splanchnic, like the development of portosystemic vessels, splenomegaly or portal hypertensive enteropathy, and extrasplanchnic, such as encephalopathy or hepato-pulmonary syndrome [2]. The partial portal vein ligation (PVL) in the rat has widely been used for the experimental study of PH since it has traditionally been accepted that portal vein stenosis does not induce liver disease [4,5].

However, we have shown that prehepatic PH by triple partial portal vein ligation (TPVL) in the rat produces microvesicular hepatocytic fatty infiltration with megamitochondria and an impairment of the liver lipid metabolism in the short (1 month) and long-term (1 year) [6-8]. Triglycerides, diacylglicerol and cholesterol increased in the liver [8]. Liver steatosis was also described in rats with PH [9] and this association was already suggested in humans over 30 years ago [10].

PH is, essentially, a type of vascular pathology related to the action of mechanical energy on splanchnic venous circulation. Mechanical energy may act in the vascular endothelium as a stressor stimulus which triggers an inflammatory response [11]. We have thus shown that in portal hypertensive-rats, splanchnic and systemic inflammatory changes are developed [12]. Portal hypertensive enteropathy mediated by mast cells, among other factors, [13] was reduced by the prophylactic administration of anti-inflammatory drugs, like Budesonide and Ketotifen [14,15]. Pro-and anti-inflammatory mediators, like NO, TNF-α, IL-1β, CO and IL-10, are released from the gut and the liver [16]. Endocrine impairments, such as increased serum levels of corticosterone and Triiodothyronine (T_3_), Thyroxin (T_4_) and decreased levels of Prolactin [17] are also shown in this experimental model. We have, therefore, proposed an inflammatory etiopathogenic hypothesis of PH [11]. Inflammation of the splanchnic system could be the key player that drives the essential nature of the complications of PH.

Although other authors described the disease as early as the 1950s [18], in 1980 Ludwig called it nonalcoholic steatohepatitis (NASH), what we now consider to be one of the manifestations of the broader nonalcoholic fatty liver disease (NAFLD) spectrum [18,19]. Although a quarter of a century later, we know that the pathogenesis of NAFLD is complex and multifactorial. Clues to its comprehension were already suggested by features highlighted by Ludwig: insulin resistance (IR) (obesity, diabetes and gallstones) fatty-inflammatory liver changes (now considered components of the endocrine system (diabetes, thyroid dysfunction and prevalence in females) [19]. Metabolic Syndrome (MS) is defined as the presence of at least three of the following changes: blood hypertension, central obesity, fasting hyperglycemia, hypertriglyceridemia and reduced high density lipoprotein (HDL) [18]. A common pathophysiological feature in this syndrome is insulin resistance [20]. However, an array of other endocrine alterations, like hypopituitarism, decreased thyroid and sex hormones, increased Glucocorticoids, the activation of Renin Angiotensin Aldosterone System (RAAS) and changes in adipokines are shown [19]. So, the link between IR, sub-clinical inflammation, MS and NAFLD is now widely accepted [19,20]. Patients with NAFLD suffer a low-grade systemic inflammatory state associated with NAFLD [21,22].

Taking all these factors in account, we can speculate that prehepatic PH in the rat could induce a type of Metabolic Syndrome related to a chronic inflammatory response. In order to verify this hypothesis we have measured the liver content of Cholesterol, Triglycerides and Phospholipids and the serum levels of Cholesterol, Triglycerides, low-density lipoprotein (LDL), HDL, Lipid binding Protein (LBP), Glucose, Insulin and Somatostatin in rats with long-term (nearly 2 years after the operation) PH.

## Results

### Splanchnic changes related to portal hypertension

The existence of portal hyperpressure in the long-term (22 months) in this experimental model of triple partial portal vein ligation was confirmed by the development of portal hyperpressure, mesenteric venous vasculopathy, splenomegaly and portal-systemic collateral circulation.

#### Portal pressure

The portal pressure (PP) in portal hypertensive-rats was higher (p = 0.004) than in sham-operated rats (Table 1).

**Table 1:** 

	**SO (n = 10)**	**PH (n = 9)**	**p value**
**PP (mmHg)**	9.25 ± 1.78	11.98 ± 1.72	0.004
**SW (g)**	1.26 ± 0.19	1.38 ± 0.35	NS
**SW/BW ×100**	0.19 ± 0.02	0.30 ± 0.06	0.0001
**BWI (g)**	401.59 ± 79.22	277.55 ± 82.95	0.004
**BWI (%)**	262.46 ± 30.62	211.02 ± 33.18	0.003
**LW (g)**	17.11 ± 2.46	12.55 ± 1.79	0.0001
**PLLW (g)**	5.84 ± 0.74	4.99 ± 0.99	0.03
**PLLW (%)**	35.76 ± 4.20	39.63 ± 4.67	NS
**ALLW (g**)	11.02 ± 1.93	7.55 ± 1.10	0.0001
**ALLW (%)**	64.24 ± 4.20	60.36 ± 4.67	NS
**LW/BW × 100**	2.62 ± 0.28	2.42 ± 0.22	NS
**TW (g)**	3.81 ± 0.35	3.22 ± 0.49	0.008
**TW/BW ×100**	0.60 ± 0.07	0.63 ± 0.16	NS

Mean ± SD; Portal pressure (PP), spleen weight (SW), spleen weight/body weight (SW/BW) ratio, body weight increase (BWI), liver weight (LW), posterior liver lobes (PLL: middle and left lateral lobes) weight; anterior liver lobes (ALL: right lateral and caudate lobes) weight, liver weight/body weight (LW/BW) ratio, testes weight (TW) and testes weight/body weight (TW/BW) ratio in sham-operated (SO) and portal hypertensive (PH) rats.

#### Mesenteric venous vasculopathy

Rats with portal hypertension showed macroscopic mesenteric vasculopathy, with dilation and tortuosity of the superior mesenteric vein and of its branches, in 77.7% (n = 7) of the animals. In 5 rats it was spontaneous (Grade II) while in 2 animals it was related to the clamping of the superior mesenteric vein (Grade I).

#### Splenomegaly

All the animals with portal hypertension showed a higher spleen weight when compared to body weight (p = 0.0001) (Table 1).

#### Collateral circulation

Interestingly enough, 22 months after the intervention all the rats presented portosystemic collateral circulation (splenorenal in 100%, hemorrhoidal in 66.6% and paraesophageal in 33.3%). Portohepatic collateral vessels through the hepatic accessory vein were shown in 33.3% of the animals.

#### Liver atrophy

Liver weight was lesser (p = 0.0001) in portal hypertensive-rats in relationship to sham-operated, but although the liver atrophy was apparent, when LW was related to body weight (LW/BW), the difference was not statistically significant. A discrete redistribution was produced in the liver mass between the liver lobes, since the weight of the posterior lobes (middle and left lateral) decreased whereas the weight of the anterior lobes (right lateral and caudate) increased (Table 1).

#### Testicular weight

Testes weight was lesser (p = 0.008) in rats with PH, but when compared to body weight (TW/BW), the results were similar in both group of rats (Table 1). So, PH in the long-term does not produce testes atrophy.

#### Body Weight

Increased body weight, even at 22 months after the intervention, was higher (p = 0.004) in sham-operated rats compared to portal hypertensive-rats (Table 1).

### Cholesterol, Triglycerides and proteins increased in the liver of portal hypertensive-rats

**Figure 1:**
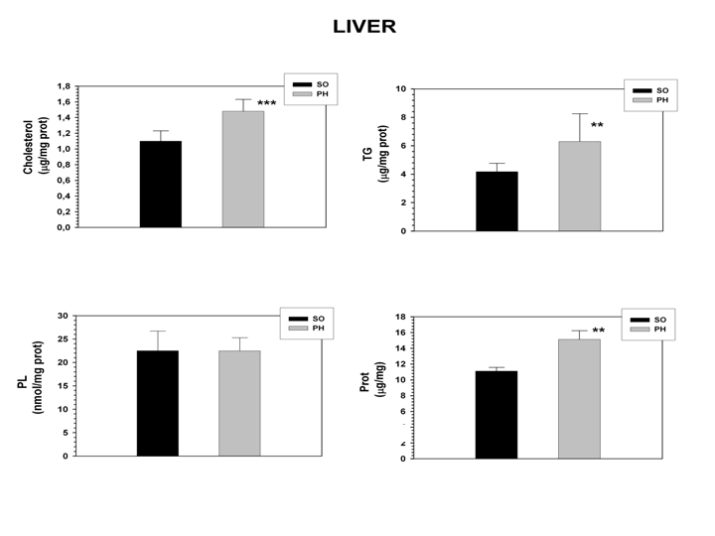
**Cholesterol, Triglycerides (TG) and Phospholipids (PL) liver content in sham-operated (SO; n = 10) and portal hypertensive (PH; n = 9) rats.** The results are represented as the mean ± the Standard Deviation. **p < 0.01; ***p < 0.001: statistically significant value in relation to the SO Group.

The liver content of Cholesterol (p < 0.001), Triglycerides (p < 0.01) and proteins (p < 0.001) increased in rats with portal hypertension compared to sham-operated rats (Figure 1).

### Serum cholesterol, Triglycerides, LDL and LBP increased and HDL decreased in rats with portal hypertension

**Figure 2:**
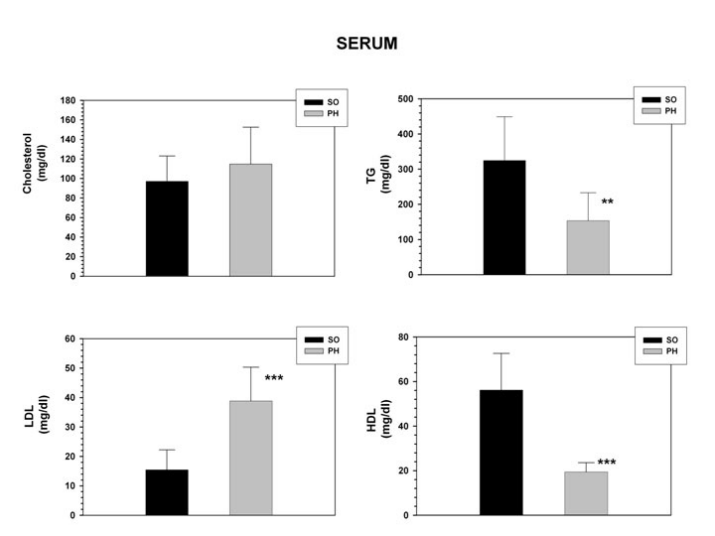
**Cholesterol, Triglycerides (TG), low-density lipoproteins (LDL) and high-density lipoproteins (HDL) serum concentrations in sham-operated (SO; n = 10) and portal hypertensive (PH; n = 9) rats.** The results are represented as the mean ± the Standard Deviation. **p < 0.01; ***p < 0.001: statistically significant value in relation to the SO Group.

**Figure 3:**
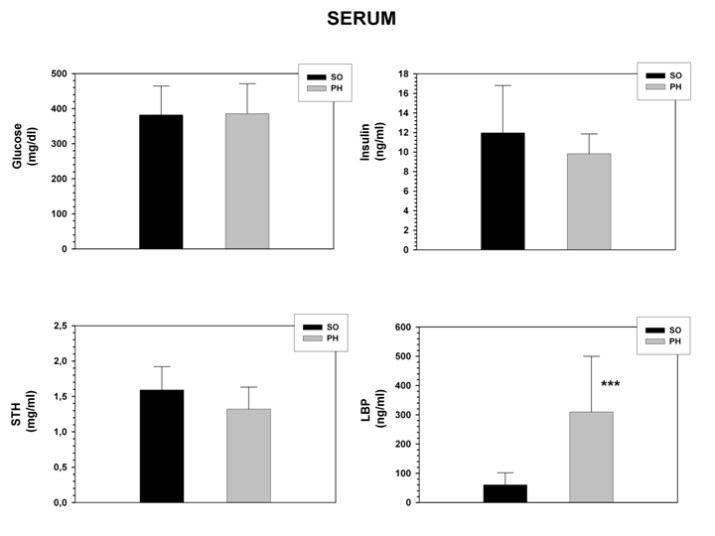
**Glucose, Insulin, Somatostatin (STH) and Lipid Binding Protein (LBP) serum concentrations in sham-operated (SO; n = 10) and portal hypertensive (PH; n = 9) rats.** The results are represented as the mean ± the Standard Deviation. ***p < 0.001: statistically significant value in relation to the SO Group.

Serum levels of Cholesterol, Triglycerides (p < 0.01), LDL (p < 0.001) and LBP (p < 0.001) increased in portal hypertensive-rats while HDL decreased (p < 0.001) (Figures 2 and 3).

### Serum Insulin and Somatostatin decreased in rats with portal hypertension

In rats with portal hypertension, serum glucose levels did not show differences in relation to sham-operated (Figure 3). On the contrary, Insulin and Somatostatin levels tended to decrease in portal hypertensive-rats (Figure 3).

## Discussion

Long-term portal hypertension (PH) in the rat shows persistent splanchnic alterations related to portal hyperpressure and produces changes in the metabolism of lipids and carbohydrates that could be involved in the development of liver steatosis, as well as in some of the manifestations described in the clinical Metabolic Syndrome (MS). Thus, in rats with PH by triple partial portal vein ligation (TPVL) we have demonstrated that triglycerides (TG) and Cholesterol increase in the liver. In the serum, levels of LDL and LBP also increase whereas TG, cholesterol, HDL and Somatostatin decrease.

Partial portal vein ligation (PVL) in various animals, but particularly in the rat, has widely been used for the study of PH [4,5]. Although this experimental model is normally used to study short-term changes (15–30 days after the operation), the study of its late evolutive phases could be considered of greater interest since the mechanisms involved in its production, as well as in the related complications, would be more similar to those found in chronic liver disease in humans [1-3] since they are related to the chronicity of PH, among other factors. In this study, the rats showed portal hyperpressure, liver atrophy, splenomegaly and portal-systemic collateral vessels. The persistence of these splanchnic changes related to PH in rats at almost 2 years (22 months) after the operation reinforces the idea that triple partial portal vein ligation in the rat is an experimental model which maintains its validity even in very late states. Interestingly enough, the mean life of male Wistar rats is approximately 2.7 years and inbred rats, like we have used in this study, indeed have shorter life spans.

We have already demonstrated in earlier (1 month and 1 year after the operation) periods, that rats with prehepatic PH by TPVL suffer a progressive liver fatty steatosis with megamitochondria formation [6,7] and an increased hepatic synthesis of free fatty acids (FFA), diacylglicerol and Triglycerides (TG) [8]. This upregulation of the lipid hepatic synthesis was associated with an increase in the liver content of cholesterol [8]. In the present study we have shown that in portal hypertensive-rats at long-term evolution, close to 2 years, the impairment of the lipid metabolism persists. Thus, these rats present an increase of liver TG and cholesterol levels, which are associated with serum lipid levels changes. Particularly, LDL and LBP increase whereas TG, Cholesterol and HDL decrease.

The sequence of events in NAFLD generally begins as hepatic steatosis, continues with steatohepatitis, and ends with cirrhosis and liver cancer [23]. Although a progressive hepatocytic fatty infiltration occurs during chronic evolution in TPVL-rats, this does not coexist with inflammation and/or fibrosis [6,8]. Therefore, hepatic steatosis developing in PH-rats could be considered a "benign" initial type of the broad spectrum of NAFLD [24].

Hepatic steatosis results from a relative increase in TG formation vs. Turnover. TG formation is, in turn, dependent on the availability of FFAs [24]. One of the new findings to emerge is that omental and mesenteric fat storage cells constitute a major endocrine influence on the liver and that the imbalance of their adipokines (e.g. leptin, adiponectin, visfatin, apelin and resistin) induces hepatic fat deposition, insulin resistance (IR) and progression to NASH [22,25]. Therefore, in rats with PH, increased lipolysis in fat-swollen mesenteric fat cells results in an especially large increase in the FFA pool entering the portal circulation and being delivered directly to the liver [26]. FFAs, after being uptaken by hepatocytes, can be oxidized as fuel [25] but when there is an excess of FFAs, they are transformed into TG, which are then stored [25]. So, high amounts of FFAs, neurohumoral and pro-inflammatory mediators, particularly TNF-a, which is increased in portal-hypertensive rats [16,27], will hit the liver inducing inflammation and steatosis [28]. Thus, TNF- a is now highlighted as a key factor in the interaction between fat storage, insulin action and inflammation[29].

However, besides the delivery of excessive FFAs from fat-swollen mesenteric and omental adipocytes and the hepato-intestinal release of pro-inflammatory mediators [7,16,30], other alterations like intestinal mucosa hypoxia with oxidative and nitrosative stress, the intestinal layer infiltration by mast cells [13] and intestinal bacterial translocation [31] stand out between the splanchnic factors probably related to the development of hepatic steatosis in rats with PH.

An increasing body of scientific research confirms the role of mastocytes in the pathogenesis of inflammatory and immunes diseases, such as the metabolic syndrome [32]. Recently metabotropic effects have been attributed to mast cells since they take part in carbohydrate and lipid metabolism [32] by decreasing the sensitivity of the liver receptors to insulin [32]. Furthermore, rat Chymase or Rat Mast Cell Protease-II (RMCP-II), which is increased in the serum and mesenteric lymph nodes in portal hypertensive-rats [15], is able to convert Angiotensinogen into Angiotensin-II (Ang II) [33]. Ang-II, in turn, initiates the gene expression of multiple pro-inflammatory mediators, interrupts the anti-inflammatory effects of Insulin [34], promotes IR [35] and stimulates the release of leptin, a prothrombotic and pro-inflammatory cytokine that activates an array of immune functions [36].

We have shown that in rats with short- and long-term portal hypertension, the balance between the different bacteria strains changes and is associated with bacterial overgrowth and BT to mesenteric lymph nodes [31]. Gut-derived endotoxin may induce steatohepatitis through adipocyte and inflammatory cells infiltrating fat increase production of Resistin, which in turn leads to IR [36]. The administration of probiotics to ob/ob mice, a model of NAFLD, led to improvement in steatosis, hepatomegaly and NF-κB activity [36,37].

When Chronic Phase Response (CFR) is developed by the body, in response to chronic stress situations, such as long-term PH, hyperlipemia, increased lipolysis of visceral fat with release of FFAs and liver IR is induced [38]. In addition, the liver increases the synthesis of Acute Phase Proteins (APP), including LPS binding protein (LBP), whose serum levels are markedly increased in rats with PH. LBP is a very sensible host mechanism that recognizes Gram-negative bacterial LPS, binds it and acts as an opsonin, enhancing bacterial phagocytosis [39]. Therefore, in chronic PH-rats, which develop a low-grade inflammatory response and which have an overgrowth of intestinal anaerobic bacteria [31], both the liver and the gut would increase the production of this important defense molecule.

In acute and chronic inflammation, infection, sepsis and MS, there is an impairment in lipid and lipoprotein metabolism, with an increase in TG, LDL and LBP and a marked decrease of HDL [40,41]. Interestingly enough, our rats with chronic portal hypertension show similar changes in lipid and lipoprotein metabolism to those presented by humans with sepsis or MS. One important original result in this study is that HDL was dramatically reduced in portal hypertensive rats and this would have important deleterious consequences. Thus, the ability of scavenging LPS by HDL would be reduced [42,43] and the anti-inflammatory and anti-oxidant properties of HDL [39,42,43] would also be inhibited.

It is now accepted that NAFLD is the hepatic manifestation of MS and it results from IR [35]. It is true that in these rats with chronic PH, serum Insulin is not increased. Indeed, Insulin levels are lesser in portal hypertensive-rats than in the sham-operated, but this could be a consequence of their chronic evolution. In NAFLD, increased FFA uptake by the liver results in hepatic glucose output, which is balanced by pancreatic islet β cells insulin production [44]. Perhaps, in these rats, a pancreatic exhaustion related to islet cell apoptosis by FFAs [44] is produced two years after the intervention and therefore β cells are unable to produce enough Insulin.

It is becoming increasingly evident that NAFLD can occur in the absence of overt IR [23]. In a similar way, the liver fat storage in rats with chronic PH accumulate lipids in their liver. This lipid storage would be mostly due to both factors, the increased support of FFAs from the inflamed splanchnic area and the inhibited FFAs liver oxidation, more than to IR.

The neuroendocrine response to stress and the existence of a systemic chronic inflammation would be another link between disordered lipid metabolism, inflammation and IR in the evolution of this experimental model. Kelley et al. [45] have proposed the idea that metabolic inflexibility, i.e., an impaired capacity to appropriately switch between "fed" (predominantly carbohydrate metabolism) and "fasting" (predominantly lipid oxidation metabolism) typifies IR and obesity. This mismatching between energy supply and demand may contribute to the accumulation of ectopic lipid, particularly in liver overtime [45]. In a similar way, the rats with long-term PH, and therefore, with subclinical chronic low-grade inflammation, inappropriately switch carbohydrate metabolism to a predominant lipid metabolism, inducing body energy imbalance and ultimately hepatic steatosis.

Thus, in long-term portal hypertensive-rats, the liver parenchyma seems to progressively convert into fat. Interestingly enough, from an embryologic point of view the liver develops in close association with the yolk sac [45] and could play a key role as an intermediary for lipid metabolism between the yolk sac and the fetus. The yolk sac is a vital player in providing lipids (cholesterol, HDL and VLDL) and lipid-soluble nutrients to the embryos during early phases of the development [46]. In experimental PH, the liver could function as a kind of yolk sac in which a pathological storage of lipids and lipid-soluble hormones and/or neuropeptides are produced in order to maintain metabolic homeostasis.

## Conclusion

Dyslipemia and hepatic steatosis would represent underlying factors of prehepatic portal hypertension in the rat which would be a chronic low-grade inflammatory state similar to that presented by the human being with acute and/or chronic inflammatory responses, i.e., infection, sepsis, NAFLD and metabolic syndrome.

## Methods

### Animals

Male Wistar rats, with average body weights of 250 g, from the *Vivarium *of the Complutense University of Madrid, were used. The animals were fed a standard laboratory rodent diet (rat/mouse A04 maintenance diet, Panlab, Spain: 17.6% proteins, 43.3% starch, 2.5% lipids) and water *ad libitum*. They were housed in a light/dark-controlled room, with an average temperature (22 ± 2°C) and humidity (65–70%) in groups of three to four animals.

All the studies were approved by the Complutense University Ethical Committee and adhered to the guidelines of the Commission Directive 86/609/EEC (*The Council Directive of the European Community*) concerning the protection of animals used for experimental and other scientific purposes. The National legislation, in agreement with this Directive, is defined in Royal Decree n° 1202/2005.

### Experimental Design

The animals were randomly divided into two groups: Group I (n = 10), sham-operated rats, and Group II (n = 9), consisted in rats with prehepatic portal hypertension by triple partial portal vein ligation (TPVL).

### Surgical Techniques

The animals were anesthetized by i.m. injection of Ketamine (100 mg/kg) and Xylacine (12 mg/kg). A midline abdominal incision was made and the portal vein was only dissected in the sham-operated (SO) animals. The surgical procedure to produce portal hypertension by triple partial ligation of the portal vein has been described previously [17]. In brief, the portal vein was isolated and three ligatures, fixed on a sylactic guide, were performed in its superior, middle and inferior portions. The stenoses were calibrated by a simultaneous ligation (4-0 silk) around the portal vein and a 20-gauge blunt-tipped needle. The midline incision was closed in two layers with an absorbable suture (Polyglycolic acid) and 3-0 silk. Analgesia was maintained during 24 hours with Buprenorphine (0.05 mg/8 h s.c.).

All the animals were sacrificed by exanguination through the inferior vena cava 22 months after the operation. Previously, the rats of both groups were anesthetized by i.m. injection of Ketamine (100 mg/kg) and Xylacine (12 mg/kg), a midline abdominal incision was made and portal pressure was registered and mesenteric venous vasculopathy and collateral circulation were studied. Finally, a sample (50 mg) of the middle lobe of the liver was rapidly frozen in acetone, chilled with dry ice and stored at -80°C until the lipid assays.

### Portal Pressure Measurement

Splenic pulp pressure, an effective indirect measurement of portal pressure (PP), was measured by inserting a 20-gauge fluid-filled needle into the spleen parenchyma. The needle was joined to a PE-50 tube, then connected to a pressure recorder (PowerLab 200 ML 201) and a transducer (Sensonor SN-844) with a Chart V4.0 computer program (ADI Instruments), and finally calibrated before each experiment.

### Mesenteric venous vasculopathy study

Mesenteric venous vasculopathy, a characteristic feature of splanchnic venous congestion, is shown as dilation and tortuosity of the superior mesenteric vein branches.

### Portosystemic collateral circulation study method

Portosystemic collateral circulation was studied as follows. First, a midline abdominal incision with a large bilateral subcostal extension was performed and then the areas in which the collateral venous circulation was developed, i.e. the splenorenal, gastroesophageal, colorectal and hepatic hilum, were carefully studied for the presence of increased collateral veins.

### Serum Assays

Blood samples were drawn by puncturing the infrahepatic inferior vena cava. After 15 minutes of centrifugation at 1,500 g, the serum was transferred to polypropylene tubes and then frozen at -42°C until lipids, lipoproteins, LBP, glucose, Insulin and Somatostatin were assayed.

The serum levels of cholesterol, Triglycerides, HDL and LDL lipoproteins were measured by spectrophotometric colorimetric techniques, according to the manufacturer instructions (SpinReact, SA, Gerona, Spain).

*Cholesterol *determination was performed with a specific kit (SpinReact) based on the action of the enzyme cholesterol sterase which hydrolyzed the esters present in the sample, giving free cholesterol and fatty acids [47]. A subsequent enzymatic oxidation using the cholesterol enzyme oxidase formed hydrogen peroxide and cholesterine. The peroxide was evaluated by the Trinder reaction by a chromogene, in the presence of peroxidase, forming a quinonimine with a red coloring. The intensity of this color was proportional to the cholesterol concentration in the sample. Finally, colorimetric determination was performed in a spectrophotometer (Eppendorf, model Biophotometer) at a wavelength of 500 nm.

*Triglyceride *determination was performed with a specific kit (SpinReact) based on the action of the enzyme lipoproteinlipase with liberation of glycerol and free fatty acids. Glycerol is converted into glycerol-3-phosphate and adenosine-5-diphosphate by glycerol kinase and ATP. Glycerol-3-phosphate is then converted by glycerol phosphate dehydrogenase into didihydroxyacetone phosphate and hydrogen peroxide. In the last reaction, hydrogen peroxide reacts with 4-aminophenazone and p-clorophenol in presence of peroxidase to give a red colored dye. The intensity of the color formed is proportional to the triglyceride concentration in the sample [48]. Finally, colorimetric determination was performed in a spectrophotometer (Eppendorf, model Biophotometer) at a wavelength of 505 nm.

*HDL-Cholesterol *determination was performed with a specific kit (SpinReact) based on the action of a product which induces the precipitation of the LDL- and VLDL-lipoproteins. Thus, the HDL-lipoproteins are only isolated in the supernatant, in which the binding of cholesterol is then measured [49]. Finally, colorimetric determination was performed in a spectrophotometer (Eppendorf, model Biophotometer) at a wavelength of 500 nm.

*LDL-Cholesterol *determination was performed with a specific kit (SpinReact), with a two-step technique. First, chylomicrons, VLDL and HDL are eliminated as cholesterol. This cholesterol, specifically derived from these lipoproteins, but not from LDL, is oxidated to colesterine and hydrogen peroxide and, then degradated to catalase. In a second reaction, LDL is specifically measured through the action of peroxidase with the formation of pinkish color quinine. The intensity of the color formed is proportional to the LDL concentration in the sample [49]. Finally, colorimetric determination was performed in a spectrophotometer (Eppendorf, model Biophotometer) at a wavelength of 600 nm.

*LBP *serum levels were assayed by the ELISA technique with a specific test for rodents (Hbt mouse LBP ELISA test kit, HyCult Biotechnology b.v., The Netherlands). The kit has a minimum detection level of 0.4 ng/ml and a measurable concentration range of 0.4 to 100 ng/ml.

*Glucose *serum levels were measured with an enzymatic technique based on the action of Glucose Oxidase (Sigma, Saint Louis, Missouri, USA). In brief, glucose is oxidized to gluconic acid and hydrogen peroxide by glucose oxidase. Hydrogen peroxide reacts with o-dianisidine in the presence of peroxidase to form a colored product. Oxidized o-dianisidine reacts with sulfuric acid to form a more stable colored product. The intensity of the pink color measured at 540 nm is proportional to the original glucose concentration [50].

*Insulin and somatostatin *were measured by RIA with our own specific antibody [51]. Tyr^2^-somatostatin was radioiodinated by the lactoperoxidase method [52]. Purification was performed using ion-exchange chromatography [53]. The sensitivity of the assay was 0.032 ± 0.002 pg/tube (n = 7). The intraassay variation ranged from 8.7% (lower part of the standard curve) to 2.1 % (middle) and 6.2% (upper). The interassay variation oscillated between 6.1 and 8.9%.

### Liver Assays

After specific extraction, TG, cholesterol, phospholipids and proteins were determined.

After the addition of 1.0 ml of chlorophorm/methanol (2:1) to the tubes containing the liver samples, the content was homogenized and centrifuged (0°C). TG, cholesterol and phospholipids were then measured by the same spectrophotometric colorimetric techniques described for the serum lipids (SpinReact, SA, Gerona, Spain).

The liver protein concentrations were determined by the colorimetric method described by Bradford [54]. This method is based on the binding of Coomassie Blue to proteins. This binding causes a displacement of the peak absorption of the dye from 465 to 595 nm. Absorbance is measured in the samples at the latter wavelength against a known reference curve. The protein complex dye has a high coefficient of extinction, which gives it a high sensitivity at measuring the protein. Protein determination was carried out because the concentration of TG, phospholipids and cholesterol is expressed in relation to the concentration of liver proteins.

### Statistical Analysis

Statistical analyses were performed using SPSS software (Statistical Package for the Social Sciences, version 14.00). The results are expressed as the mean ± standard deviation. Student t-test for independent data were used for comparison of the variables between the two groups studied. The results were considered significant if p < 0.05.

## Abbreviations

APR: acute phase reaction; APP: acute phase protein; Ang-II: angiotensin-II; CFR: chronic phase response; CO: carbon monoxide; FFA: free fatty acids; HDL: high density lipoproteins; IL: interleukin; IR: insulin resistance; LBP: lipid binding protein; LDL: low density lipoproteins; MS: metabolic syndrome; NAFLD: non-alcoholic fatty liver disease; NASH: non-alcoholic fatty liver disease; NF-κB: factor nuclear-κB; NO: nitric oxide; PL: phospholipids; PH: portal hypertension; PVL: partial portal vein ligation; RAAS: renin angiotensin aldosterone system; RMCP-II: rat mast cell protease; T_3_: triiodothyronine; T_4_: thyroxin; TG: triglycerides; TNF-α: tumor necrosis factor α; TPVL: triple partial portal vein ligation; VLD: very low density lipoproteins.

## Authors' contributions

FSP, MPN and MAA performed most of the experiments and provided assistance for the preparation of the manuscript. EV, CG and RA carried out the laboratory assays. MAA, EV and JA participated in the design of the study and prepared the manuscript. All authors have read and approved the content of the manuscript.
